# Case Report: Role of hypoglycemia in seizure aggravation in a case of focal epilepsy: revealing a missing link between diabetes and dementia

**DOI:** 10.3389/fnins.2025.1604552

**Published:** 2025-06-27

**Authors:** Hiroya Ohara, Masami Yamanaka, Kiyoko Inoue, Hironori Shimizu, Naohiko Iguchi, Keiko Tanaka, Abilash Valsala Gopalakrishnan, Balachandar Vellingiri, Masako Kinoshita

**Affiliations:** ^1^Department of Neurology, Minaminara General Medical Center, Nara, Japan; ^2^Department of Clinical Laboratory, Minaminara General Medical Center, Nara, Japan; ^3^Department of Clinical Laboratory, Yoshino Hospital, Nara, Japan; ^4^Department of Neurology, Nara Medical University School of Medicine, Nara, Japan; ^5^Department of Animal Model Development, Brain Research Institute, Niigata University, Niigata, Japan; ^6^Department of Biomedical Sciences, School of Bio Sciences and Technology (SBST), VIT, Vellore, Tamil Nadu, India; ^7^Department of Zoology, Central University of Punjab, Bathinda, Punjab, India; ^8^Department of Neurology, National Hospital Organization Utano National Hospital, Kyoto, Japan

**Keywords:** Alzheimer’s disease, Brief Potentially Ictal Rhythmic Discharges, electroencephalography, flash glucose monitoring, focal epilepsy, hypoglycemia, long-term video-electroencephalography monitoring, temporal lobe epilepsy

## Abstract

**Aim:**

While low-frequency electroencephalographic (EEG) activity increases during hypoglycemia, the relationship between hypoglycemia and changes in epileptic activities has not been fully investigated. Recently, the American Clinical Neurophysiology Society’s EEG Terminology 2021 defined criteria for Brief Potentially Ictal Rhythmic Discharges (BIRDs) including rhythmic fast activities. We evaluated the association between hypoglycemia and BIRDs.

**Methods:**

Data from a 27-year-old female with focal epilepsy and idiopathic hypoglycemia, who underwent scalp-recorded long-term video-EEG using the International 10—20 system with T1/T2 electrodes, were analyzed. Her anti-neuronal antibody test results were negative. EEG recordings over 6 h were retrospectively evaluated in longitudinal bipolar montages at 15 s per display screen. The number and duration of BIRDs were assessed in each 30 min epoch. Glucose levels were obtained using a flash glucose monitoring system, and the average glucose level for each epoch was calculated using the area under the curve (AUC), measured by pixel-counting software. The relationship between the number and duration of BIRD subtypes and average glucose levels was evaluated using cut-off values of 70, 60, and 50 mg/dL.

**Results:**

During the recording, the EEG showed focal slow activities, epileptic spikes, and BIRDs in the left temporal area, but no clinical or electrographic seizures were observed. The number of evolving BIRDs per epoch was significantly higher during more severe hypoglycemia when the cut-off values were set at 60 mg/dL (2.00 ± 0.71 vs. 0.38 ± 0.70, mean ± SD, *p* < 0.05, Mann–Whitney U test) and 50 mg/dL (2.33 ± 0.47 vs. 0.44 ± 0.68, *p* < 0.05). The total duration of definite BIRDs per epoch also showed a statistically significant difference when the cut-off was set at 50 mg/dL (3.15 ± 1.82 vs. 2.10 ± 1.00 s, *p* < 0.05).

**Conclusion:**

Maintaining glucose levels above 60 mg/dL appears important for the early termination of epileptic rhythmic discharges. Individuals with diabetes are at high risk of Alzheimer’s disease (AD), and hippocampal hyperactivity contributes to epileptic seizures, amyloid deposition, and disease progression. Fluctuations in blood glucose levels, including episodes of hypoglycemia, increase the risk of dementia. The present findings suggest a potential causative role of hypoglycemia in AD and propose a precise method to correlate glucose levels with brain activities.

## Introduction

Since Hans Berger’s first observation, it has been well established that the frequency of background EEG activity decreases during hypoglycemia (Berger, 1937). Hypoglycemia is a major cause of acute symptomatic seizures, with a proposed threshold of 36 mg/dL (Beghi et al., 2010). A recent case report described an 11-year-old boy with type 1 diabetes who developed drug-resistant epilepsy and neurological sequelae following hypoglycemia at 35 mg/dL (Sokolewicz et al., 2024). Neonatal hypoglycemia has also been associated with structural brain damage and the subsequent development of epilepsy (Bartolini et al., 2023; Fong and Harvey, 2014). However, it remains unclear whether hypoglycemia can induce changes in rhythmic fast activities—potentially of epileptic origin—and exacerbate seizures in patients with epilepsy.

Recently, flash glucose monitoring has been employed to continuously track interstitial glucose levels, showing good correlation with capillary blood glucose measurements (Bailey et al., 2015). For EEG evaluation, the American Clinical Neurophysiology Society’s Standardized Critical Care EEG Terminology: 2021 Version (ACNS2021) introduced precise criteria for categorizing electrographic and electroclinical seizures, as well as Brief Potentially Ictal Rhythmic Discharges (BIRDs)—rhythmic fast activities that do not meet criteria for electrographic seizures but are associated with epileptic events (Hirsch et al., 2021). The integration of these tools enables a detailed evaluation of the temporal relationship between blood glucose fluctuations and brain activity.

In this study, we investigated the association between hypoglycemia and BIRDs in a patient with focal epilepsy and idiopathic hypoglycemia, using simultaneous flash glucose monitoring and long-term video-EEG.

## Case description

A 27-year-old female patient who had experienced clonic convulsions 3 years earlier and subsequently developed recurrent focal seizures—manifesting as nausea, convulsions, and retrograde amnesia—was evaluated. Antiseizure treatment with levetiracetam (1,500 mg) and lamotrigine (250 mg) provided partial symptom control; however, her seizures worsened over the last 2 months.

Her medical history included ulcerative colitis, ovarian endometriotic (chocolate) cysts, Meniere’s disease, insomnia, and depression. Her treatment regimen comprised flunitrazepam 2 mg, trazodone hydrochloride 150 mg, vonoprazan fumarate 10 mg, eszopiclone 3.0 mg, ramelteon 8.0 mg, risperidone 2.0 mg, lemborexant 10 mg, alprazolam 0.8 mg, quetiapine 200 mg, prednisolone 12.5 mg, and methylcobalamin 1,500 μg. Her family history included panic disorder and type I diabetes mellitus with high anti-glutamic acid decarboxylase antibody titers in her mother, and lung cancer in her father.

Blood testing revealed hypoglycemia (49 mg/dL), but no underlying cause was identified despite extensive evaluation; therefore, a diagnosis of idiopathic hypoglycemia was made. EEG revealed intermittent irregular delta waves and epileptic spikes localized to the left temporal region. Cerebrospinal fluid analysis was unremarkable, and tests for oligoclonal bands and anti-neuronal antibodies—including anti-glutamic acid decarboxylase, N-methyl-D-aspartate receptor, leucine-rich glioma-inactivated 1 protein, contactin-associated protein 2, *α*-amino-3-hydroxy-5-methyl-4-isoxazolepropionic acid receptor, *γ*-aminobutyric acid receptor type B, dipeptidyl-peptidase-like protein-6, and glycine receptor antibodies—were all negative. Brain MRI showed no abnormalities.

To assess the potential contribution of hypoglycemia to seizure exacerbation, the patient underwent simultaneous long-term video-EEG and flash glucose monitoring.

This study was conducted in accordance with the principles of the Declaration of Helsinki, and the case report was prepared in compliance with the CARE guidelines. Approval of the study design was waived by the Institutional Ethics Review Board of Minaminara General Medical Center based on its classification as a retrospective analysis of anonymized clinical data. Written informed consent was obtained from the patient. Part of this study was presented in abstract form at the XXVI World Congress of Neurology in 2023, held in Montreal (Ohara et al., 2023).

## Diagnostic assessment

**Figure 1:**
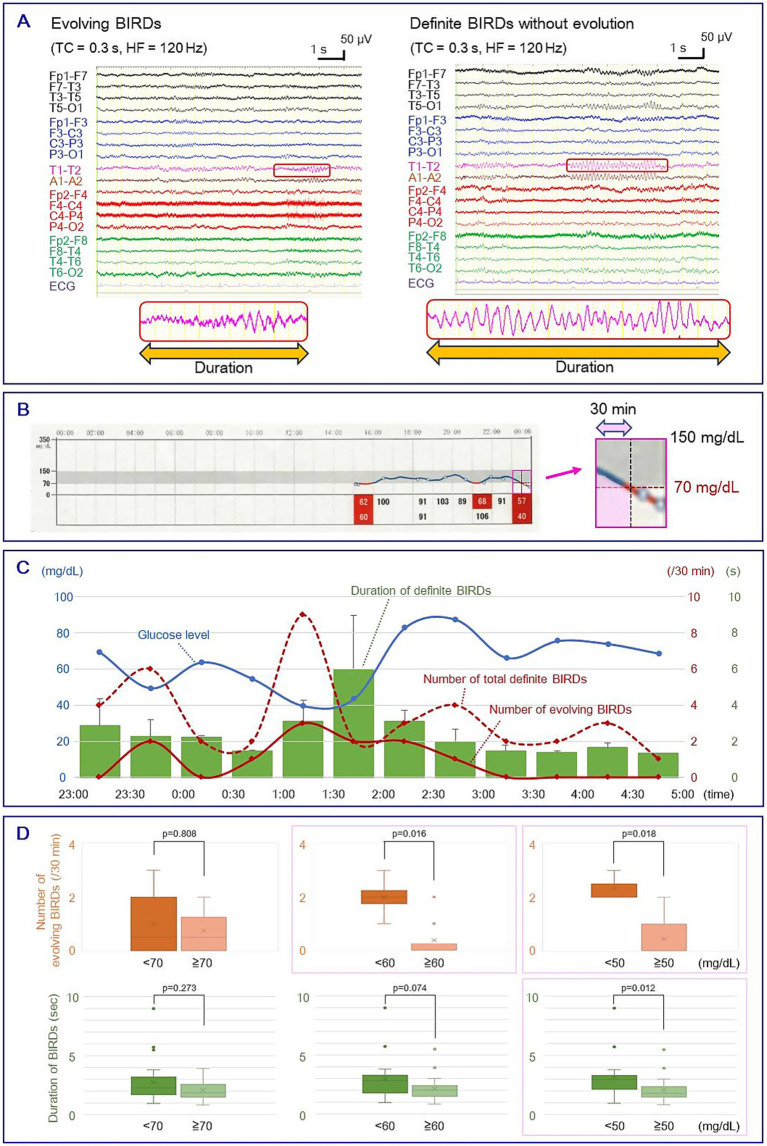
**(A)** Left: evolving BIRDs; Right: non-evolving definite BIRDs. Enlarged waveforms at the bottom show the duration of BIRDs. **(B)** Measurement of average glucose level. Left: raw graph of glucose levels obtained by the flash glucose monitoring system. The gray area indicates glucose levels between 70 and 150 mg/dL, and the pink square shows a 1 h segment. Right: An enlarged image of the 1 h segment showing the measurement of the AUC of a 30 min epoch (pink area). **(C)** Temporal changes in glucose level (blue line), number of total definite BIRDs (red line), number of evolving BIRDs (red dotted line), and duration of BIRDs (green bars). Error bars indicate standard deviation. **(D)** Box-and-whisker plots of the number of evolving BIRDs (upper row) and the duration of definite BIRDs (lower row) with cut-off glucose levels of 70, 60, and 50 mg/dL. Boxes represent the median and interquartile range (IQR). Whiskers indicate the maximum and minimum values within 1.5 × IQR, and outliers are shown as dots. Mean values are indicated by crosses. The number of evolving BIRDs was significantly larger in severe hypoglycemia when cut-off values were set at 60 mg/dL (2.00 ± 0.71 vs. 0.38 ± 0.70, mean ± SD) and 50 mg/dL (2.33 ± 0.47 vs. 0.44 ± 0.68) (*p* < 0.05, Mann–Whitney U test), and the duration of total definite BIRDs showed a significant difference when a cut-off value was set at 50 mg/dL (3.15 ± 1.82 vs. 1.97 ± 0.96 s) (*p* < 0.05) (pink squares). BIRDs: Brief Potentially Ictal Rhythmic Discharges. AUC, area under the curve.

Long-term video-EEG was recorded using an EEG 1224 (Nihon-Kohden Tokyo, Japan) via scalp electrodes placed according to the International 10–20 system with T1/T2. The sample rate was 500 Hz, and the low-cut filter was 0.51 Hz (time constant: 0.3 s). Glucose levels were obtained using the FreeStyle Libre Flash glucose monitoring system (Abbott Japan LLC, Tokyo, Japan). To avoid the effects of food intake, consecutive EEG recordings with simultaneous glucose measurements for 6 h during the nighttime (23:00–05:00) were analyzed. EEG data were retrospectively reviewed by two board-certified clinical neurophysiologists (H. O. and M. Y.) in a longitudinal bipolar montage with T1–T2 and A1–A2 derivations at 15 s per display screen. In each 30 min epoch, rhythmic fast activities satisfying the focal BIRDs criteria according to ACNS2021 were identified (Figure 1A). In brief, definite BIRDs were defined as rhythmic activities above 4 Hz, consisting of at least six waves, lasting 0.5 to 10 s, and either evolving (evolving BIRDs, a form of definite BIRDs) or occurring at the same location as interictal spikes (non-evolving definite BIRDs) (Hirsch et al., 2021). Possible BIRDs, which were sharply contoured but did not meet the criteria for definite BIRDs, were excluded. Considering the delay between interstitial and blood glucose levels, the area under curve (AUC) for each 30 min epoch was measured using pixel-counting software, and the resulting value was used as the average glucose level during that epoch (Figure 1B). We evaluated the relationship between the number and duration of BIRDs and average glucose levels by setting cut-off values of 70, 60, and 50 mg/dL. As the datasets violated the assumption of normality, non-parametric tests were employed to evaluate statistical significance. The significance level was set at *p* = 0.05 for group comparisons. All statistical analyses were performed using SPSS Statistics 27.0 J (IBM Japan, Tokyo, Japan).

Figure 1C shows the temporal changes in glucose levels and the characteristics of BIRDs. During the recording, the EEG showed focal slow activities, epileptic spikes, and BIRDs in the left temporal area, but no clinical or electrographic seizures were observed. Of the 40 definite BIRDs analyzed, 11 were evolving BIRDs and the remaining 29 were non-evolving BIRDs. The number of evolving BIRDs per epoch was significantly higher in more severe hypoglycemia when the cut-off values were set to 60 mg/dL (2.00 ± 0.71 vs. 0.38 ± 0.70, mean ± SD, *p* < 0.05, Mann–Whitney U test) and 50 mg/dL (2.33 ± 0.47 vs. 0.44 ± 0.68, *p* < 0.05) (Figure 1D; Supplementary Table 1). The duration of total definite BIRDs per epoch showed a statistically significant difference when a cut-off value of 50 mg/dL was applied (3.15 ± 1.82 vs. 2.10 ± 1.00 s, *p* < 0.05) (Figure 1D; Supplementary Table 1). No statistically significant difference was found using a cut-off value of 70 mg/dL.

By precisely controlling glucose levels while maintaining antiseizure medications, she achieved seizure freedom for more than 4 years.

## Discussion

Our patient showed hypoglycemia-associated EEG changes using simultaneous recording of video-EEG monitoring and flash glucose monitoring. Considering that BIRDs are closely associated with electrographic seizures, clinical epileptic seizures, and medically refractory epilepsy, and that the evolving pattern is one of the most important components for qualifying electrographic seizures (Hirsch et al., 2021), the EEG findings of the current study suggest that patients with focal epilepsy can become more susceptible to seizures as glucose levels decrease. Our findings provide new insight into the mechanism by which even mild hypoglycemia can trigger hippocampal hyperactivity and lead to neuronal damage.

In this study, a glucose cutoff value of 60 mg/dL showed a significant difference in BIRDs, which was higher than the threshold for acute symptomatic seizures (36 mg/dL) (Beghi et al., 2010). Although there is wide inter-individual variability in EEG responses to glucose levels, our patient demonstrated that seizure threshold lowering had already begun when the glucose level fell below 60 mg/dL. A previous study that evaluated cortical excitability using transcranial magnetic stimulation showed significantly decreased long intracortical inhibition during fasting compared to the postprandial state, with glucose levels ranging from 88.2 mg/dL to 145.8 mg/dL in patients with generalized epilepsy, and from 95.4 mg/dL to 142.2 mg/dL in those with focal epilepsy (Badawy et al., 2013). Thus, daily fluctuations in blood sugar levels—even within the normal range—can exacerbate neuronal hyperexcitability in patients with epilepsy who are prone to seizures.

Additionally, focal seizures originating in the temporal lobe, as in our patient, often involving the hippocampus, are more vulnerable to hypoglycemia than those arising from other brain regions. An animal model study in rabbits demonstrated that hypoglycemic electrical seizures consistently originated in the amygdala or hippocampus (Tokizane and Sawyer, 1957). In humans, Green evaluated EEG changes during tolbutamide-induced hypoglycemia as a potential activation method to detect cerebral lesions in 60 adult patients with various neurological disorders and found that seizures were induced in 32 (53%) patients, half of whom had focal seizures, with epileptiform EEG abnormalities most prominent in the temporal areas (Green, 1963). Among them, a 43-year-old man who complained of brief attacks of painful paresthesia in his right arm and leg for 2 months developed a focal tonic seizure on the right side lasting 8 s during induced hypoglycemia at a blood glucose level of 55 mg/dL. Ictal EEG showed bitemporal 4 Hz activity followed by background suppression. Pneumoencephalogram revealed a marked midline shift, and brain surgery confirmed a grade 2 astrocytoma in the left temporal lobe. A more recent case report described a 61-year-old diabetic patient who developed a focal seizure of temporal origin due to insulin-induced hypoglycemia at 46 mg/dL, with clear EEG documentation showing 5–6 Hz rhythmic slow waves involving the right temporal region (Lapenta et al., 2010). With the application of the new criteria for definite BIRDs, we were able to precisely evaluate EEG changes in relation to blood glucose levels.

Epilepsy is closely associated with dementia. The Framingham Heart Study demonstrated a bidirectional association between epilepsy and dementia, indicating that the presence of either condition doubles the risk of developing the other (Stefanidou et al., 2020). In Alzheimer’s disease (AD), Aβ accumulation leads to abnormal synchronization in the hippocampal circuitry, network hypersynchrony, and seizures (Palop and Mucke, 2016; Sen et al., 2018). Hippocampal hyperexcitability, not only during overt clinical seizures but also during subclinical epileptiform activity—such as spikes and sharp waves in individuals without a clinical epilepsy diagnosis—accelerates Aβ release, amyloid deposition, and disease progression in a vicious cycle (Palop and Mucke, 2016; Sen et al., 2018; Noebels, 2011; Vossel et al., 2016; Babiloni et al., 2025). Additionally, EEG data from AD patients with subclinical epileptiform discharges show abnormalities in physiological oscillatory rhythms, which are essential for the regulation and maintenance of vigilance, even during recordings without overt epileptiform activity (Babiloni et al., 2025).

Epileptic seizures and dementia share common vascular risk factors (Sen et al., 2018). Individuals with diabetes—a well-established vascular risk factor—are at increased risk for AD and related dementias (Biessels et al., 2006). A recent study found that individuals with focal epilepsy are four times more likely to develop dementia (hazard ratio [HR] 4.02, 95% confidence interval [CI] 3.45–4.68), and those with both focal epilepsy and high cardiovascular risk (including diabetes and *APOE* e4 allele) are over 13 times more likely to develop dementia (HR 13.66, 95% CI 10.61–17.60) (Tai et al., 2023). In type 2 diabetes, high or unstable HbA_1c_ levels are associated with a greater risk of dementia (HR 1.08, 95% CI 1.07–1.09 per 1% HbA_1c_ increment; HR 1.03, 95% CI 1.01–1.04 per 1 SD increment, respectively) (Zheng et al., 2021). Hypoglycemia significantly contributes to dementia. Severe hypoglycemia episodes leading to hospitalization or clinical attention during both mid-life (age 45–64 years) and late-life (age 65–84 years) are associated with increased dementia risk (HR 2.85, 95% CI 1.72–4.72 and HR 2.38, 95% CI 1.83–3.11, respectively) (Alkabbani et al., 2023). Underwood et al. recently evaluated the association between HbA_1c_ stability and dementia incidence using the HbA_1c_ time in range (TIR), demonstrating that a TIR < 20% (compared to ≧80%) increased the incidence of dementia (HR 1.23, 95% CI 1.19–1.27) and that ≧60% time below range (compared to ≧60% TIR) also increased dementia risk (HR 1.23, 95% CI 1.19–1.27) (Underwood et al., 2024). Severe hypoglycemia can cause brain damage, including neuronal death, gray matter loss, and cortical atrophy (Suh et al., 2007), through mechanisms such as amyloid genesis, tau phosphorylation, and induction of a proinflammatory state (Lauretti and Praticò, 2015; Yang et al., 2016). Furthermore, imbalances in glucose homeostasis can disrupt signaling pathways involved in neurogenesis and synaptic plasticity, contributing to neurodegeneration (Stranahan et al., 2008; Mainardi et al., 2015).

This study had several limitations. First, the data were obtained from a single case, limiting generalizability. Second, this was an observational study without a control group or intervention, preventing the establishment of a strong causal link. Third, the patient was on multiple medications, some of which could have influenced EEG patterns or glucose metabolism. While antiseizure medications are known to suppress epileptic discharges, lamotrigine may suppress wide-band power at high doses, and levetiracetam typically produces no significant change or only minor increases in beta-band power (Veauthier et al., 2009; Duveau et al., 2016). Benzodiazepine hypnotics and tranquilizers such as flunitrazepam and alprazolam are known to increase beta-band activity (Tan et al., 2003; Saletu et al., 1994). Ramelteon, a melatonin receptor agonist, enhances fast gamma oscillations in the rat primary motor cortex during non-REM sleep (Yoshimoto et al., 2021). Risperidone, an antipsychotic, can reduce alpha and beta band power (Maxion et al., 2024; Dimpfel, 2007). Exogenous corticosteroids have been shown to increase theta activity in healthy individuals, correlating with mood and cognitive changes (Wolkowitz, 1994). However, considering the pharmacokinetics of these medications, it is unlikely that the 30 min interval EEG fluctuations observed in this study were caused by these drugs. Future accumulation of similar cases will help improve generalizability and facilitate real-world applications of these findings.

### Patient perspective

The present observations indicate that maintaining glucose levels above 60 mg/dL is important for the early termination of epileptic activity. Special caution should be exercised when managing patients with temporal lobe seizures, as this region appears particularly susceptible to hypoglycemia-induced excitability. These findings also support a potential causative role of hypoglycemia in the development of AD and related dementias. Furthermore, this study proposes a precise approach for correlating glucose levels with brain activity, offering new insights into metabolic influences on neuronal excitability.

## Data Availability

The original data presented in this study are included in the article and supplementary material. Further inquiries can be directed to the corresponding author.
