# Supplementary material for: Case Report: Role of hypoglycemia in seizure aggravation in a case of focal epilepsy: revealing a missing link between diabetes and dementia
Source: Front Neurosci. 2025 Jun 27;19:1604552. doi: 10.3389/fnins.2025.1604552 (PMC12245777; doi:10.3389/fnins.2025.1604552)
Supplement: Supplementary file 1 [file Data_Sheet_1.pdf]

Supplementary Table 1. Temporal changes in glucose level, number of total definite BIRDs, number of evolving BIRDs, and duration of BIRDs

Time	Glucose level (mg/dL)	Number of total definite BIRDs	Number of evolving BIRDs	Duration of definite BIRDs (s, mean ± SD)
23:00-23:30	69.3	4	0	2.88 ± 1.53
23:30-0:00	49.3	6	2	2.27 ± 0.94
0:00-0:30	63.7	2	0	2.23 ± 0.10
0:30-1:00	54.5	2	1	1.47 ± 0.02
1:00-1:30	39.5	9	3	3.11 ± 1.72
1:30-2:00	43.5	2	2	5.98 ± 3.01
2:00-2:30	82.8	3	2	3.12 ± 0.61
2:30-3:00	87.3	4	1	1.95 ± 0.76
3:00-3:30	66.2	2	0	1.48 ± 0.32
3:30-4:00	75.5	2	0	1.41 ± 0.08
4:00-4:30	73.8	3	0	1.68 ± 0.25
4:30-5:00	68.5	1	0	1.34 ± 0.00

BIRDs: Brief Potentially Ictal Rhythmic Discharges.
